# Transition of Care for Individuals with Mental Disorders in Brazil: A Contextual Analysis

**DOI:** 10.1590/0034-7167-2023-0063

**Published:** 2023-12-04

**Authors:** Larissa Arielly Cunha da Silva, Renilly de Melo Paiva, Sarah Lyandra Furtado Faustino, Emanuela Juliana Bezerra Miranda, Viviane Euzébia Pereira Santos

**Affiliations:** I Universidade Federal do Rio Grande do Norte. Natal, Rio Grande do Norte, Brazil

**Keywords:** Mental Health, Transitional Care, Mental Disorders, Brazil, Patient Safety, Salud Mental, Cuidado de Transición, Transtorno Mental, Brasil, Seguridad del Paciente, Saúde Mental, Cuidado Transicional, Transtorno Mental, Brasil, Segurança do Paciente.

## Abstract

**Objective:**

To describe the contexts of care transition for individuals with mental disorders in the Brazilian setting.

**Methods:**

A contextual analysis was conducted through a scoping review. The search for studies was conducted in databases and thesis and dissertation portals, and the analysis was based on immediate, specific, general, and meta-contexts.

**Results:**

The sample, consisting of eight studies, indicated that the following factors are present in the contexts where care transition occurs: Peculiarities of care transition for individuals with mental disorders; Perspectives that can strengthen or weaken this transition; Approaches proposed in the past for the development of care transition; and Elements related to Brazilian legislation.

**Final Considerations:**

It is observed that the transition of care for individuals with mental disorders in Brazil takes place in various contexts of care levels. These variations present significant potentials and barriers in the care scenarios.

## RESUMO

**Objetivo:**

Descrever os contextos de transição do cuidado de pessoas com transtorno mental no cenário brasileiro.

**Métodos:**

Análise de contexto realizada por meio de *scoping review*. A busca por estudos foi feita em bases de dados e portais de teses e dissertações, e a análise baseou-se nos contextos imediato, específico, geral e metacontexto.

**Resultados:**

A amostra, composta por oito estudos, apontou que, dentre os fatores presentes nos contextos em que ocorre a transição do cuidado, destacam-se: particularidades da transição do cuidado a pessoas com transtorno mental; perspectivas que podem fortalecer ou enfraquecer essa transição; abordagens propostas no passado para o desenvolvimento da transição do cuidado; e elementos relacionados às legislações brasileiras. **Considerações fina**is: Percebe-se que a transição do cuidado a pessoas com transtorno mental no Brasil ocorre em diversos contextos dos níveis de atenção. Essas variações apresentam potencialidades e barreiras significativas nos cenários de cuidado.

## RESUMEN

**Objetivo:**

Describir los contextos de transición del cuidado de personas con trastorno mental en el escenario brasileño.

**Métodos:**

Análisis de contexto realizado a través de una revisión exploratoria (scoping review). La búsqueda de estudios se llevó a cabo en bases de datos y portales de tesis y disertaciones, y el análisis se basó en los contextos inmediato, específico, general y metacontexto.

**Resultados:**

La muestra, compuesta por ocho estudios, señaló que, entre los factores presentes en los contextos donde ocurre la transición del cuidado, se destacan: particularidades de la transición del cuidado a personas con trastorno mental; perspectivas que pueden fortalecer o debilitar esta transición; enfoques propuestos en el pasado para el desarrollo de la transición del cuidado; y elementos relacionados con la legislación brasileña.

**Consideraciones finales:**

Se percibe que la transición del cuidado a personas con trastorno mental en Brasil ocurre en diversos contextos de los niveles de atención. Estas variaciones presentan potencialidades y barreras significativas en los escenarios de cuidado.

## INTRODUCTION

The subject of this study is the transition of care for individuals with mental disorders in Brazil. The anti-asylum movement granted new rights to these individuals, challenging the notion that they should be isolated and hospitalized. As such, health services restructured the mental health care model with the goal of integrating these individuals into various care contexts. However, the transition from one service to another needs to be articulated and communicated.

A mental disorder is characterized by significant changes in an individual’s cognition, emotional regulation, and/or behavior. These changes can reflect their biological, psychological, and/or mental development processes. They can affect social and professional activities and are often associated with distress or disability^(1)^.

This condition severely impacts the global population and is considered one of the causes of disability at work, with its prevalence gradually increasing over time. It is estimated that 450 million people worldwide suffer from mental disorders, and in Brazil, the prevalence is around 20% among adults^(2)^. About 36 million Brazilians suffer from illnesses such as depression; globally, this number approaches 350 million people^(3)^.

Care for these patients goes beyond dehospitalization and sees the family as a crucial link in the process, being the primary support network for the individual^(4)^. Aiming to redirect the care model for these people in Brazil, services such as Primary Health Care (PHC) were designed to promote care based on the needs of this population. To achieve this, it is essential to have health professionals trained for collaborative, multiprofessional care, aiming to develop integrative practices in PHC^(5)^.

The beginning and continuity of this care become a challenge for health systems worldwide due to the range of professionals operating in various health services where care processes occur. These professionals need to be trained and sensitized to the demands of each patient and for the continuity of care, without disruptions between one service and another. Therefore, it is essential to promote care transition^(6)^.

The transition of care is the foundation for the continuity of assistance and one of the main strategies linking healthcare networks^(7)^. In the transition process, actions include discharge planning, patient and family health education, coordination between health services, communication between the team and various patient referral services, and post-discharge follow-up^(6)^. Thus, the role of nursing in caring for individuals with mental disorders goes beyond biological and technical actions; it should prioritize interpersonal relationships, welcoming attitudes, and encourage the patient’s active participation in their own care^(8)^.

The care transition process can occur between various sectors of the same institution, such as from a hospital to an outpatient clinic, primary care, or even to the home. This coordination between services is achieved through notifications about the patient’s discharge to the reference service, either by telephone or other communication methods, made from the hospital to PHC, or through electronic records/systems. Regardless of the method used, this care needs to be planned and agreed upon with the patient, family, and involved services^(9)^.

To better understand and recognize how transitional care develops for patients with mental disorders, the guiding question is defined as: in what contexts does the transition of care for individuals with mental disorders occur in Brazil?

## OBJECTIVE

To describe the care settings of transition in care for people with mental health disorders in the Brazilian scenario.

## METHODS

### Ethical Aspects

Since this is a review study, there was no involvement of human beings, which waives the need for approval by a Research Ethics Committee.

Theoretical-methodological Framework This is a context analysis according to the proposal by Hinds, Chaves, and Cypess (1992), where context is defined as a set of four layers interacting in the face of a fact or scenario: immediate context, specific context, general context, and meta-context. The immediate context focuses on the present moment when the act occurs; the specific context is characterized by the present elements that will influence the phenomenon; the general context encompasses past and current interactions that will influence the phenomenon; and the meta-context emphasizes the past and present, also highlighting alternatives for the future^(10)^.

### Type of Study/Methodological Procedures

This contextual analysis was structured based on the criteria established by COnsolidated criteria for REporting Qualitative research - COREQ^(11)^ for qualitative research. For the construction and analysis of contexts, a search was conducted in July 2022 in national and international databases, as well as in the grey literature, using the keywords: 1) Mental health disorders, 2) Transition in care, 3) Brazil.

### Collection and Organization of Results

The eligibility criteria for material included publications that addressed the objective of the study and were fully available through the *CAFe* protocol electronically. Excluded were studies in the form of editorials, letters to the editor, and opinion articles. These criteria were applied to address the proposed research question and the study’s objective so that they addressed the care settings of transition in care for patients with mental health disorders in the Brazilian scenario.

There were no language restrictions or time limits, and duplicate studies were only considered once. The selection of studies was conducted by a team of trained peer reviewers independently for material collection, reading, and study selection, in two steps: reading the title and abstract, followed by a full reading of the selected studies.

The collection took place in the PubMed, CINAHL, SCOPUS, COCHRANE databse, Web of Science, Psychinfo, LILACS, ERIC database, CAPES Theses and Dissertations Portal, The National Library of Australia’s Trove (Trove), Academic Archive Online (DIVA), DART-Europe E-Theses Portal, Electronic Theses Online Service (EThOS), Open Access Scientific Repository of Portugal (RCAAP), National ETD Portal, Theses Canada, and Theses and Dissertations of Latin America databases. The search was done in pairs, and in the event of a disagreement, a third collector was consulted.

The considered variables were: care settings, types of transitions that occur between services, and the main findings (benefits/challenges). From the analysis of the initially found 965,270 studies, eight matched the final sample.

**Figure 1:**
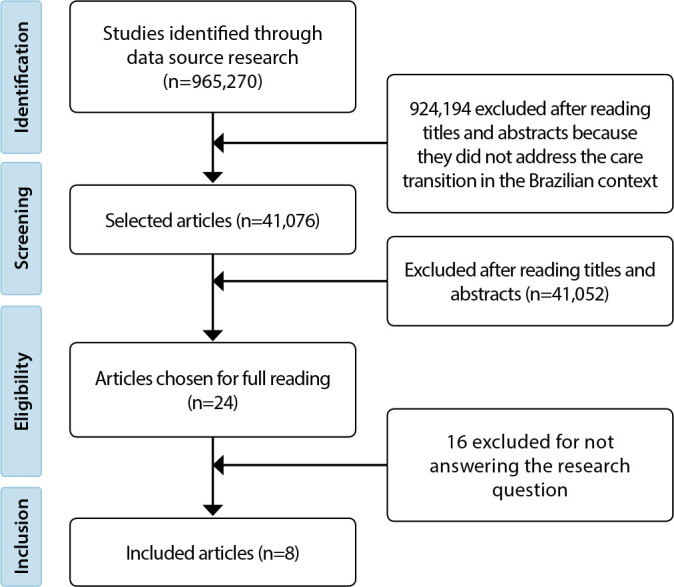
Study selection stages (N=8), 2022

The study selection is shown in the following flowchart (Figure 1).

Thus, for this study, the immediate context will be the direct characteristics of care transition for individuals with mental disorders in Brazil; the specific context will be interpretations of actions taken or not, which facilitate or hinder care transition; the general context will encompass relevant aspects from the past and measures that have been proposed or taken for the development of care transition for individuals with mental disorders; and the meta-context will refer to elements related to laws and regulations in Brazil.

### Data analysis

After setting the references for analysis, a thorough reading of all the selected material was conducted, distinguishing the elements that characterize each publication (database, language, year of publication, country, study objective, methodological design, evidence level), in addition to information regarding transitional care (care contexts, types of transitions occurring between services and sectors, main findings on benefits and challenges). From the analysis, the data were presented descriptively and grouped according to each specific context level, described, and represented graphically with the intention of relating them.

## RESULTS

The selected studies were published between 2000 and 2021. Regarding the types of transitions that occur between services and sectors, four (50%) of the studies addressed the care transition process starting from the hospital level to other services, whether home-based or Psychosocial Care Network (RAPS in Portuguese). The other four (50%) studies showed variations in the onset of this transitional care from sectors such as Primary Health Care (PHC), Mental Health Reference Centers (CERSAM in Portuguese), or Psychosocial Care Centers (CAPS in Portuguese), as shown in Chart 1.

**Chart 1:** Synthesis of care settings and types of transitions that occur between health services (N=8), 2022

Type of Material	Title	Care Settings	Types of Transition Between Services
Article	The family and the mentally ill user of the day-hospital - a case study^(12)^	Hospital-based	Day hospital to Home
Article	Mental health and continuity of care in health centers in Belo Horizonte, MG^(13)^	Secondary care	CERSAM to Health Centers
Article	Admission criteria and continuity of care in psychosocial care centers, Rio de Janeiro^(14)^	Secondary care	CAPS to PHC or outpatient clinic
Article	Day hospital and psychosocial care center: Expanding the discussion of partial hospitalization in mental health^(15)^	Hospital-based	Day hospital to CAPS
Article	Psychiatric admissions and re-admissions in a general hospital in Porto Alegre: sociodemographic, clinical characteristics, and the use of the Psychosocial Care Network^(16)^	Hospital-based	Hospital to RAPS services (CAPS, outpatient clinic, PHC)
Article	Performance of Psychosocial Care Centers in four urban centers in Brazil^(17)^	Secondary care	PHC to CAPS and hospital
Article	Care for individuals with mental distress in primary care: an interdisciplinary and multiprofessional practice^(18)^	Primary care	PHC to Home
Article	Transitions of care in mental health^(19)^	Hospital-based	Hospital to PHC

The selected studies addressed different methodological aspects that show the variation of methods in which transitional care can be applied, as shown in Chart 2.

**Chart 2:** Summary of the methodological aspects of the selected studies (N=8), 2022

Reference	Design	Type of Study	Method	Data Collection Instrument	Analysis Method
Monteiro; Barroso^(12)^	Qualitative	Descriptive	Case study	Semi-structured interviews	Content analysis
Oliveira; Caiaffa; Cherchiglia^(13)^	Quantitative	Descriptive	Retrospective cohort study	Medical records	Content analysis
Cavalcanti et al.^(14)^	Qualitative	Descriptive	Participatory evaluation	Records, team information, patient and family information	Content analysis
Weber; Juruena^(15)^	Qualitative	Technical Documentation	Documentary research	Review of Brazilian legislation on mental health	Content analysis
Zanardo et al.^(16)^	Quantitative	Descriptive and Analytical	Observational, cross-sectional study	Semi-structured interviews	Content analysis
Onocko-Campos et al.^(17)^	Quantitative	Descriptive	Cross-sectional	Semi-structured interviews	Content analysis
Almeida et al.^(18)^	Qualitative	Descriptive	Prospective cohort study	Semi-structured interviews	Content analysis
Bueno et al.^(19)^	Quantitative	Descriptive	Retrospective cohort study	Semi-structured interviews	Content analysis

In relation to the contextual levels^(10)^, the immediate context identified the direct specifics of the care transition for individuals with mental disorders in Brazil. Examples include: coordination and assistance of health professionals, patients’ connection to the service, logistical arrangements, regularity and continuity of treatment, and education for both the patient and their family.

In the specific context, highlighted perspectives can either strengthen or weaken the care transition. Among the facilitating factors are: interaction, family coexistence, substitute services, therapeutic planning, standardized information transfer, welcoming procedures, and systematic care. On the other hand, obstacles include hospital readmissions, lack of communication between the team and the patient, and scattered, isolated services.

In the general context, approaches from the past for the development of care transition in Brazil are evident, such as referring patients to services through a reference and counter-reference process with feedback. Matrix support is also emphasized as a powerful tool for professionals, envisioning a more integrated approach to encourage interdisciplinary practice and better care for patients with mental disorders.

The meta-context encompasses elements related to Brazilian legislation. The guidelines and recommendations from the National Law of Psychiatric Reform and the Unified Health System (SUS) notably influence the National Mental Health Policy (PNSM in Portuguese), the Psychosocial Care Network (RAPS), the Psychosocial Care Centers (CAPS), the Therapeutic Residence Services, and the National Patient Safety Program. Together, they provide a foundation for implementing and continuing transitional care practices for individuals with mental disorders.

**Figure 2:**
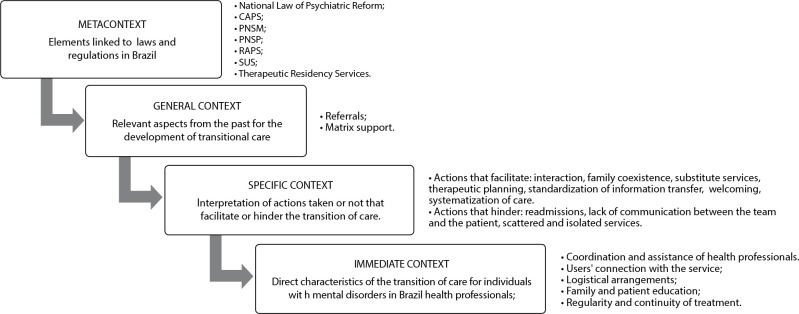
Contexts of care transition for individuals with mental disorders in Brazil, 2022

Thus, the contextual levels are as follows: the direct characteristics of care transition for individuals with mental disorders in Brazil (immediate context); interpretations of actions taken or not, which facilitate or hinder the care transition (specific context); significant aspects from the past and measures that have been proposed or undertaken for the development of transitional care (general context); and elements related to laws and regulations in Brazil (meta-context), as illustrated in Figure 2.

*CAPS: Psychosocial Care Centers; PNSM: National Mental Health Policy; PNSP: National Patient Safety Policy; RAPS: Psychosocial Care Network, SUS: Brazilian Unified Health System.*

## DISCUSSION

### Meta-context - Elements related to laws and regulations in Brazil

The implementation of laws, policies, and ordinances aimed at promoting quality of life and reducing vulnerabilities and inherent health risks of people with mental disorders, in addition to ensuring care and continuity across sectors, is regarded as a landmark for the health of these individuals. The laws establishing SUS and the Psychiatric Reform aim to ensure universality, comprehensiveness, and equity in access and assistance to the population. They also seek to protect the rights of individuals with mental disorders by redirecting the care model.

With the creation of SUS, mental health became part of the comprehensive care system, implementing new care models through policies and ordinances. Notable among these are the RAPS, the CAPS, and the Therapeutic Residence Services within the SUS framework. These ensure coordination and integration of care points within the network, aiming to promote the social reintegration of users. This process was strengthened based on post-positivist relations established with patients with mental disorders, characterized by a hospital-centric paradigm, where the emphasis was placed on the disease rather than the individual^(20)^.

It’s also worth noting the National Mental Health Policy and the National Patient Safety Policy, which emerged to redirect the care model and contribute to the quality of health care in all establishments across the country, following a global trend. This prompted the organization of care networks ranging from primary, secondary, and tertiary services, to providing assistance based on the needs of each individual^(21)^.

### General Context - Relevant historical aspects for transitional care development

From a general perspective, several historical aspects have contributed to the process of facilitating care transitions for individuals with mental disorders. Among these are the use of referrals and matrix support. Health sectors, whether in primary, secondary, or tertiary care, often operate with patient referral flows (reference) to other services or receipt of patients (counter-reference). However, studies have identified flaws in this process which, consequently, disrupt treatment continuity, leading to negative impacts on users, such as care discontinuation, increased suicide rates, and frequent readmissions^(13)^.

Issues such as unclear operational guidelines, inadequate infrastructure, and a lack of service planning and evaluation are considered major barriers to care transition. This is underscored by the fact that many patients are referred to health centers without their medical records or follow-up reports. Hence, there is a clear weakness in the care line, given that effective communication between services is often lacking^(13)^.

To ensure genuine communication and mitigate structural disorganization within the service network, strategies for professional operation were proposed and implemented. Matrix support, established in 2004 by the Ministry of Health, became an enabling strategy aimed at connecting Basic Health Units with mental health services. The interdisciplinary integration of professionals catalyzes changes in patient care, interventions, planning, and enhances cohesion between primary care and reference services^(18)^.

### Specific Context - Interpretation of actions taken or not, which facilitate or hinder care transition

In this light, activities that either support or hinder care transition stand out. These include interaction with professionals, family life, systematization of care, communication failures, and scattered and/or isolated services. Among the actions promoting care transition are alternatives to psychiatric hospital admission, such as CAPS III and Day Hospitals for mental health, recognized for their diverse intervention methods and synergistic operation. As a result, there is an emphasis on fostering and valuing interaction and family coexistence, which ease the adaptive process for patients^(12-15)^.

However, inadequate care transitions can impair communication between patients and health professionals. It is emphasized that therapeutic planning and standardized information transfer facilitate communication, reception, systematic assistance, and action management to support safe care for this population^(19)^.

Issues concerning infrastructure, service planning, and evaluation, along with unclear operational guidelines, impact the transition and continuity of patient treatment. Therefore, a weakened health system and a lack of care coordination lead to frequent readmissions^(13-16)^.

### Immediate Context - Direct Characteristics of Transitional Care for Individuals with Mental Disorders in Brazil

Improving access to primary care is essential, as the majority of patients diagnosed with a mental disorder are assessed by professionals in this sector. In this light, studies have pointed to the emergence of RAS, strategies aimed at integrating services. They are crucial in overcoming care gaps and optimizing available resources^(19-22)^.

To achieve this, logistical arrangements in health systems are vital. Based on the operational structure of RAS, the goal is to integrate sectors in response to fragmented care and to lessen the multiplicity of concepts, principles, and dimensions. Therefore, coordinating mental health care ensures that users’ needs are met across articulated sectors, reinforcing patient safety^(21)^.

Considering the need for care during crises, suicide attempts, and/or social vulnerability, service provision to these individuals aligns with logistical systems. This is exemplified by services provided by the Psychosocial Care Network in sectors such as CAPS, general hospitals, emergency services, clinics, among others^(21)^.

Grounded in ensuring better reception to users, the coordination and assistance developed by the multiprofessional team is emphasized. It focuses on bonding between the team and service users, educating patients and families, and maintaining treatment regularity and continuity^(12,15,17-19)^.

### Study Limitations

It is highlighted that the contextual analysis has limitations due to the few studies found on the subject. This did not allow for a more in-depth delineation on the matter. The research was limited to the Brazilian scenario, considering the unique context of health in Brazil. Therefore, there is a suggestion for more in-depth investigations on the national scene and future studies that consider the international scene for detailed analysis.

### Contributions to the Fields of Nursing, Health, or Public Policy

By mapping the main places where care transitions for individuals with mental disorders occur and identifying relevant aspects in each contextual layer, it is possible to elucidate the elements influencing the phenomenon in Brazil. This broadens the view on the care transition for these patients. For nursing, such mapping contributes to systematic care where professionals can understand and intervene in the services’ reality and guide the population to services based on their needs. Thus, the provided information, grounded in scientific evidence, offers insights for new approaches by the professional team to patients with mental disorders and enhances the care delivered.

## FINAL CONSIDERATIONS

The transition of care for people with mental disorders in Brazil occurs in hospital, primary, and secondary care settings. It is done between hospital care environments to home or RAPS sectors (PHC, CAPS, clinics), and/or among these network sectors working interconnectedly. Such care variations highlight the existence of actions that can either facilitate or hinder the development of transitional care. Therefore, it is pertinent to conduct and disseminate more studies on the subject that address professionals’ practical experiences and devise strategies in health sectors to strengthen the care transition for patients with mental disorders.
